# Supplement to Reports on Continued Fever

**Published:** 1852-01

**Authors:** 


### BUFFALO MEDICAL JOURNAL

AND

### MONTHLY REVIEW.

VOL. 7. JANUARY, 1852. NO. 8.

### ORIGINAL COMMUNICATIONS.

### ART. I.—SUPPLEMENT TO REPORTS ON CONTINUED FEVER.

By the Editor.

*Symptoms distinctive of Typhoid and Typhus Fever. Identity of these two types. Diagnosis, etc.*

In the preceding investigations, Continued Fever is considered under two forms, or types, distinguished by the names *Typhus* and *Typhoid.* It has been a leading object in these investigations to study the symptoms, etc., belonging to each of these types, separately, and by comparison as respects the results developed by analytical examination. Connected with this nosological subdivision of Continued Fever are several questions of interest and importance, the discussion of which would have been out of place in Reports devoted to historical facts, but which invite supplementary consideration. These questions pertain to—1st. The propriety of the division into *Typhus* and *Typhoid* fever, and the validity of the distinctive symptoms upon which that divison is based; 2d. The essential identity or non-identity of *Typhus* and *Typhoid* fever, and the relationship existing between the two types; 3d. The diagnostic traits involved in the discrimination of *Typhus* and *Typhoid* fever from each other, and of Continued Fever from other affections.

What views relating to the foregoing subjects are most consonant with the results and conclusions set forth in the two Clinical Reports that have preceded? In the few remarks which will follow, I purpose to confine myself, mainly, within the scope of this inquiry, not professing to measure the range of discussion by the amplitude of the subjects, nor undertaking to collect from available sources all that has an important bearing thereupon.

A brief sketch of the *symptoms distinctive of Typhus and Typhoid fever* which are embraced in the two Reports, will form a proper preliminary to a consideration of the several points just named. In order to study the phenomena of the two types separately, and comparatively, it was, of course, necessary, as a primary step, to make the distinction of type, and arrange the cases in corresponding groups. In so doing, it is obvious that the existence of symptoms distinctive of either type is predetermined, and the results of the comparison of the two types, in this respect, in a measure forestalled. The application of rules for discriminating between the two types is thus to be made, in collecting facts which are to test the correctness of these rules. This was unavoidable, but it will be found to have had its advantages, showing in how far obvious external distinctive symptoms are practicably available for the discrimination. In classifying cases in order to bring the results of the analysis of each type into comparison with those of the other, it is evident that accuracy of discrimination is indispensable. Unless cases which are grouped as *Typhus* and *Typhoid* are correctly collocated, the conclusions cannot fail to be erroneous. With reference to this point, as stated in the Reports, all cases in which there appeared any room for uncertainty respecting the type to which they belonged, were classed by themselves, under the head of *cases of Doubtful type.* In this way, it is presumed, the liability to error on the score just mentioned, was obviated. In making the discrimination practically, those rules were followed which are laid down by late writers, and more especially in the able and lucid treatise by Prof. Bartlett.

In proceeding to give, succinctly, the more important of the facts, contained in the two Reports, which appear to present traits distinguishing the two types from each other, it will be convenient to follow an order of arrangement corresponding with the successive sections in both Reports.

SUMMARY OF SYMPTOMS DISTINCTIVE OF TYPHOID AND TYPHUS FEVER.

*Age.* The maximum, and mean age higher in *Typhus* than in *Typhoid.*

*Nativity.* In *Typhoid*, patients foreign immigrants from different countries, with a certain proportion of citizens of this country. In *Typhus*, foreign immigrants except when the disease is communicated by contagion, and almost invariably the immigrants recently from Ireland.*

*Season.* The liability to *Typhoid* much greater during the autumnal months; *Typhus* equally and even more apt to occur during other portions of the year.

*Duration of disease prior to admission into hospital.* In a marked degree shorter in *Typhus* than in *Typhoid.*

*Symptoms of access. Diarrhœa* present in a certain proportion of cases in *Typhoid* during the access, not in *Typhus.*

*General aspect.* Capillary congestion of the face, extending frequently to the extremities, and more or less over the body, causing a dull red color, present in *all* cases of *Typhus*, and in a large proportion of the cases of *Typhoid;* but the congestive redness greater in *Typhus* than in *Typhoid*, and presenting in some cases of the former type a dusky or dingy hue.

NERVOUS SYSTEM. *Passive delirium*, manifested by incoherent talking, muttering, attempting to get out of bed, etc., present more constantly in *Typhus;* developed earlier in the progress of the febrile career in *Typhus;* but very active, persistent delirium, requiring forcible restraint, character stic of *Typhoid.*

*Cephalalgia*, oftener present after the fever becomes established, and longer in duration in *Typhoid*, (? see second Report.)

*Vascular injection of conjunctiva* oftener present in *Typhus*, (? see first Report.)

*Deafness*, present in a proportion of cases larger in *Typhus.*

DIGESTIVE SYSTEM. *Appetite*, or relish of food, oftener present in *Typhus*, (see second Report.)

*A reddened tongue*, occasionally observed in *Typhoid*, and not in *Typhus.*

*Sordes* present in a larger proportion of cases of *Typhus.*

*Vomiting*, more apt to occur in *Typhoid.*

---

* It will be borne in mind that this summary embraces the conclusions deduced from the facts contained in the previous Reports. Were it extended so as to embrace conclusions deduced from facts contained in other analyses, it would not only comprehend additional distinctive symptoms, but some of those which it embraces might require qualification. This note is suggested by what is stated under the head of nativity.

*Diarrhœa*, present in *one-half* of the cases of *Typhoid*, and in *one-third* of the cases of *Typhus;* in the latter type always mild or slight, but in the former sometimes prominent as a symptom.

*Hæmorrhage from the bowels*, characteristic of *Typhoid.*

*Tympanites*, present in about an equal ratio in both types; but in *Typhus* almost invariably slight, while in *Typhoid* it is often prominent.

*Tenderness on pressure over the abdomen*, an almost constant symptom in *Typhoid*, and less frequently present in *Typhus*. In the latter usually slight, and in the former more apt to be marked, or considerable in degree.

*Peritonitis from perforation of intestine*, peculiar to *Typhoid.*

*Eruption.* An eruption present in a larger proportion of cases of *Typhus;* more abundant in *Typhus*, frequently very copious, and extending over the upper extremities, as well as on the trunk; in *Typhoid* seldom copious, often only sparely scattered over abdomen and chest, and not present in the extremities. The characters of the eruption as follows: In *Typhoid*, rose colored, oval, slightly elevated, redness momentarily disappearing on pressure. In *Typhus*, of a dull red color, smaller in size, not elevated, redness imperfectly disappearing on pressure. These characters preserved in the two types in the great majority of cases. Occasionally slight variations, and some intermingling of the two kinds of eruption.

Respiratory System. *Cough* more uniformly present in *Typhus* (?)

*Pneumonitis* more apt to be developed in *Typhus* (?)

*Epistaxis* extremely rare in *Typhus*, occurring frequently in *Typhoid.* Sputa detached from the posterior nares apt to be tinged with blood in *Typhoid*, not in *Typhus.*

Circulation. Greater frequency of the pulse in *Typhus*, than in *Typhoid.* The average frequency in the former exceeding an hundred per minute, in the latter falling below that number. Instances of great increase in frequency occurring oftener in *Typhus.*

Skin, exclusive of eruptions and congestive redness, not presenting symptoms distinctive of either type.

*Duration*, longer, before death, or convalescence, in *Typhoid*, than in *Typhus*, this rule being subject to variations, owing to an unusually short duration of the *Typhoid* type at some periods. (See second Report.)

The foregoing summary contains several striking points of contrast pertaining to the symptomatology of the two types of Continued Fever, corresponding in the main with facts heretofore contributed by different observers. In no important particulars do the results of the preceding analyses directly conflict with those which furnished the distinguishing traits of *Typhus* and

*Typhoid* fever as laid down in the treatise by Bartlett and some other systematic works. In so far as these results go, they concur in sustaining the correctness of this nosological division of Continued Fever, and exemplify the distinctive symptoms upon which it rests.

It is, however, to be borne in mind, that in arranging the cases for analysis, a certain number were classed under the head of *Doubtful type;* and the question arises, why were not the symptoms distinctive of *Typhus* and *Typhoid* applicable to these, as well as to the others? This is a question not to be overlooked, but it will be more appropriately considered in connection with the subject of *diagnosis,* and is, therefore, for the present deferred.

The lesions found after death in the two types supply important distinctions. But aside from these, the propriety of the nosological division seems sufficiently established; and they will be more especially involved in the subject next to be considered.

### IDENTITY OF TYPHUS AND TYPHOID FEVER.

It has been, of late years, a mooted queston, even with those who recognize the propriety of the nosological division of Continued Fever into *Typhus* and *Typhoid*, whether these two types are essentially identical, or not. What relationship do they sustain toward each other? Are they simply varieties of one disease, or are they distinct affections? These inquiries claim a few remarks.

In the summary of distinctive traits which has been presented, facts appear to show the existence of different laws as respects *age*, *season*, *duration*, and various *symptoms* pertaining to the several anatomical systems in which these symptoms are distributed. These facts concur with those developed, in the same manner, on a more extended scale, by other analyses. The latter, moreover, appear to show additional laws pertaining to the causation, transmission by contagion, etc., which it does not fall within my present plan to consider, having restricted myself, mainly, to the results of the analytical investigation of the limited number of cases embraced in my own collections.

Both *Typhus* and *Typhoid* may be considered, in a measure, eruptive fevers, and in all eruptive affections, difference in the characters of the eruption, provided it be marked, and constant, is regarded as denoting a difference in the character of the disease. Now, the characters belonging to the *Typhus* and *Typhoid* eruptions are by no means identical. The resemblance is hardly greater than between Scarlatina and Rubeola in this respect. As a general rule, also, it would seem that the eruption peculiar to either type maintains its distinctive characters, but this rule is not invariable. An eruption is occasionally observed presenting a combination of the characters belonging to both types. Such instances are probably few, and perhaps do not oftener occur, and not to a greater extent than in measles and scarlet fever. In this connection it may be remarked that the fact of the distinctive traits pertaining to the eruption, and other symptoms, having been so recently pointed out, cannot be considered as evidence against the non-identity of *Typhus* and *Typhoid* fever, for, with respect to Rubeola and Scarlatina, although no one now regards them as merely different varieties of the same disease, it is but little more than half a century since they were so regarded, and at a period not very remote small pox was included in the same category.

The lesions found after death have an important bearing on the present subject. On behalf of the non-identity of the two types, it is claimed that *Typhoid* fever is characterized by special morbid changes seated in the aggregated and solitary follicles of the small intestines, accompanied by enlargement of the mesenteric glands. The spleen, also, is said to be generally enlarged and softened. These lesions, more particularly the intestinal, it is stated, are peculiar to *Typhoid* fever, being uniformly found after death, in cases proving fatal, and not found in bodies dead with other acute diseases. In *Typhus*, on the other hand, these lesions, especially those noted in the follicles, are declared to be invariably absent.

On this subject the preceding Reports contain the results of a few observations, upon which, as yet, no remarks have been offered, notice of them having been reserved for this connection. Of the fatal cases, the bodies were examined with particular reference to the lesions deemed to be characteristic of *Typhoid* fever in *twelve*. Of these cases, *nine* had been classed under the head of *Typhoid*, judged by the symptoms, and *three* under the head of *Typhus*.

On reference to the description of the morbid appearances in these cases, severally, it will be seen that in each of the *nine* *Typhoid* cases there existed notable changes in the intestinal follicles accompanied by corresponding degrees of enlargement of the mesenteric glands. The follicular lesions consisted either of considerable tumefaction, rendering the patches very prominent and salient, due manifestly to a deposit of some morbid material in these parts, the surfaces being a more or less roughened or presenting a granular appearance, in other words ulcerated; or there existed in the sites of the follicular patches depressed spaces which presented in some instances an ulcerated aspect, and in other instances a smooth healthy surface. The latter appearance seemed to have resulted from an elimination of the morbid material producing the appearances first mentioned, a kind of sloughing, with a corresponding loss of substance, or ulcerated excavations, which subsequently healed. I have observed, although I have failed to record this fact, the sloughing process just referred to, partially completed in some patches, a portion of the patch being excavated, and the remainder still occupied with a material apparently in the process of separation.

The changes in the spleen were less constant. This organ, however, as is well known, varies considerably in bulk within the limits of health, and alterations in consistence are less appreciable than in other parts of greater normal firmness of structure.

In each of the *three Typhus* cases, on the other hand, there existed changes in the follicular patches, accompanied, in *two* cases, by very slight enlargement of the mesenteric glands. The follicular lesions, however, were insignificant in comparison with those observed in the *Typhoid* cases. The patches were simply developed so as to be visible. They were slightly hypertrophied, not projecting several lines above, or depressed below the level of the mucous surface, as in the *Typhoid* cases, and in no instance presenting an ulcerated aspect. The contrast, indeed, was scarcely less striking than if, in the *Typhus* cases, the follicles had remained invisible.

The united number of observations, thus, which are contained in the preceding Reports sustain the existence of characteristic lesions of the intestinal follicles and mesenteric glands in *Typhoid* fever, but they also show that these parts do not always continue wholly unaffected in *Typhus*. The fatal cases of the latter type, in which post mortem examinations were made, are few in number, but they are sufficient to establish the fact that the intestinal follicles and mesenteric glands may be, to some extent affected in that type, provided it be certain that the diagnosis of the cases was correct, and of this fact ample evidence is adduced from the histories in connection with the descriptions of the post mortem appearances.

As before stated, the present scope of inquiry does not embrace a collection and critical examination of all the available facts bearing on the point under consideration which have been contributed by different writers. It is well known, however, to those at all conversant with the literature of the subject, that several distinguished observers have testified to the invariable absence of the abdominal lesions of *Typhoid* fever in numerous cases falling under their observation in which, during life, were presented the symptoms distinctive of *Typhus*. The most remarkable testimony of this description is by Dr. Gerhard, of Philadelphia, to whom, more than any other individual, is due the merit of having pointed out the distinguishing features belonging to *Typhus*, contrasted with *Typhoid*, derived from the study of the symptoms during life, as well as the post mortem appearances. In over fifty autopsies he found no deviation from the normal condition of the Peyerian glands except in a single instance, and that case the diagnosis* was doubtful.

On the other hand, cases have been reported in which the follicular glands of the intestine have presented more or less alteration in cases in which, during life, the peculiar eruption, and other symptomatic characters of *Typhus* were observed. The most remarkable testimony of this description is by M. H. Landouzy, of France, who affirms that in an epidemic at the prison of Rheims, during which one hundred and thirty-eight cases occurred, the histories of which embraced the characteristic eruption and other distinctive features of *Typhus*, six autopsies were made, and the elliptical plates of the ilium, in all, found to be either thickened, elevated, or *ulcerated*.† Prof. Bartlett, who advocates the opinion that the two types are radically distinct affections, admits that the paper of M. Landouzy throws some doubt on the subject. Without having seen the original paper it would be unfair to offer criticisms, or to conjecture sources of error. The remark, however, may be admissible, that in accepting results which, in the present position of the subject, are so important in their bearings, it should be understood, in the first place, that the symptomatology of the cases was deduced from recorded data, and more especially that the histories of the six fatal cases referred to were noted at the bed side; and, in the second place, that the appearance of the intestinal lesions were fully described. In the absence of that confidence arising from personal knowledge of the historian, the above precautions are requisite as a guaranty, on the one hand, that, in the epidemic described, cases of both types of Continued Fever were not intermingled and confounded, and, on the other hand, that the follicular intestinal changes were not within the limits in which, as must be admitted, they may be present in fatal cases of well marked *Typhus*. From the abstract of the paper given in the treatise of Bartlett, it does not appear whether the account of the epidemic is based on an analysis of recorded observations, or not. By these remarks I would by no means be understood to express the opinion that observations must necessarily be recorded to merit any confidence, or possess value. Such an opinion would be as absurd, as that all observations which are recorded, as a matter of course, are reliable and important. The idea intended to be conveyed is, that every provision for accuracy should be

* Am. Jour. Med. Sciences, 1837.

† Bartlett's Treatise, from *Archives Generales de Medicine*. 1842.

required before accepting as valid, observations which conflict materially with other well-authenticated facts, and in the correctness of which highly important principles are involved.

The following extract from an article contained in the British and Foreign Medico-Chirurgical Review, [No. for July, 1851, Am. Edition, page 26,] gives a fuller account of the facts reported by M. Landouzy, than I have met with elsewhere. The article referred to had not appeared when the foregoing remarks were written. The reader will doubtless concur in the opinion expressed by the reviewer in the closing sentence of the extract:

"Seventeen cases died, of whom six only were examined, *and only two cases are recorded.* The first case was indubitably typhus; the mulberry rash was well marked. Landouzy says he 'looked in vain for the rose-spots of typhoid fever which existed among other patients.' (p. 313.) Nevertheless, at the necropsy he found alteration of Peyer's patches. But after careful examination of the report, we have great doubt on this head. The small intestines were first examined by M. Chabaud and two other physicians, and *pronounced to be sound;* then M. Landouzy entering and re-examining it, detected what he considered evidence of incipient change in Peyer's patches, with general slight tumefaction of the solitary glands. But we doubt whether M. Landouzy has made out his case; the alterations he signalizes are not sufficiently defined to bring it within Louis' definition of typhoid fever.*

Now, since these are the only two recorded cases, and neither of them is satisfactory, and since only four other post-mortem examinations were made, the details of which are not given, and the value of which is, therefore, necessarily shaken by the exception we must take to the two recorded cases, we do not think we can do otherwise than consider that the importance of this memoir of M. Landouzy has been very much overrated, and that the facts recorded in it cannot be received without the greatest doubt, as evidence on this point.† We feel no hesitation, then, in putting this memoir aside, as being indefinitely precise, and based on far narrower details than the writings on the other side of the subject."

That the intestinal follicles may become affected in some manner, and to a greater or less extent, in *Typhus*, is certain. But it is, perhaps, a question yet remaining to be settled definitively, whether in *Typhoid* fever these parts are not the seat of *lesions of a special character* which are never found in *Typhus.* Putting aside the paper of M. Landouzy, I cannot refer to any observations showing the existence of lesions corresponding to those presented

* The appearances Landouzy describes are perfectly well known to the German observers as dependent on a "catarrhal condition" of the intestinal mucous membrane, which may occur in typhus, as in pneumonia, or any acute disease. No accurate observer could confound this with typhoid deposit in and under Peyer's patches.

† Landouzy himself perceives so many differences between typhus and typhoid fever, that in spite of his belief that he had found the anatomical sign of typhoid in typhus, he writes: "I find between the typhus of Rheims, and the typhoid fever, too striking differences to allow me to regard these two maladies as identical." (p. 329.) He says, however, that they are analogous, but not identical.

in the nine fatal *Typhoid* cases the histories of which, with the autopsies, are embraced in my collections, when it was perfectly clear, *from the symptoms noted at the bed side*, that the disease was of the *Typhus* type. I do not affirm that no such observations have been contributed, but only that, if they exist, I am unable to refer to them, not professing to have bestowed, in connection with this discussion, sufficient search to warrant a positive assertion that they are not to be found.

Another question arises in connection with the subject of the abdominal lesions in Continued Fever. It is this: — Assuming that changes characteristic of *Typhoid* fever exist, are they uniformly present in cases of that type? They are, of course, only susceptible of demonstration in cases proving fatal, by examinations after death. Is more or less disease in the follicles and mesenteric glands an essential constituent of *Typhoid Fever?* This question involves a correlative inquiry, viz., are cases of Continued Fever in which the follicles are unaffected necessarily cases of *Typhus?* These questions are important, but the Reports to which these remarks are supplementary, do not contain any facts bearing upon them. Louis, whose researches established the significance of these lesions as the "*anatomical characteristic*" of *Typhoid* fever, evidently formed the conclusion that they constituted an essential element of the disease, before the analysis of the cases in his collection was completed, for he excludes cases in which were present the important symptoms of the disease, but in which the intestinal lesions were absent. He classes such cases under the head of "*Simulated cases of Typhoid affection.*" In the remarks succeeding the history of one of the cases under this caption, he holds the following language: "This fact presents, as we see, the union of almost all the characteristic symptoms of the *Typhoid* affections. At the commencement there was diarrhœa, alternating with drowsiness and delirium; shortly after there were pains of the abdomen, more or less decided meteorism, which continued until death; after these, eschars on the sacrum and great trochanters, and return of delirium. *What more was wanting to make us certain that the patient was affected with the Typhoid disease, and that we should find, on opening the corpse, the elliptical patches of the small intestine more or less seriously altered? Notwithstanding this, the patches and the corresponding mesenteric glands were healthy, and with the exception of a small extent of the mucous membrane of the colon, the whole intestinal canal was natural, and how, then, can we admit that this patient had the Typhoid affection.*" *

* Louis, on Fever. Translated by H. I. Bowditch, M. D., vol. ii, page 371.

It is clear from the above quotation, a portion of which I have italicized, that, after proceeding a certain length in his investigations, Louis fixed upon the intestinal lesions as a constant element of the disease. At whatever period in his researches this conclusion was formed, it would necessarily follow that all subsequent cases of *Typhoid* fever proving fatal would have the lesion, since if the lesion was absent, the disease was not to be considered *Typhoid* fever! Plainly, in prosecuting a series of autopsical examinations with a view to settle the point of the constancy of special changes in a disease, the presence of the disease must have been determined, in order to avoid a *petitio principii*, by other criteria than the changes which are the objects of search.

If this criticism be just, it follows, that a case exhibiting "an union of almost all the characteristic symptoms of the *Typhoid* affection," and in which nothing "more was wanting to make us certain that the patient was affected with the *Typhoid* disease" than that the elliptical patches should be found affected, must be regarded as neither more nor less than a case of the *Typhoid* affection, as was pronounced prior to the autopsy. Other cases detailed by Louis in the same category, as cases of simulated *Typhoid* affection, are perhaps to be considered veritable cases of this disease equally with that to which reference has just been made. Assuredly, it is by the ensemble of symptoms during life that the determination of the type of fever is to be made, not to say that in fatal cases more or less importance does not belong to the lesions, either in confirming or correcting the diagnosis. A post mortem test, it is obvious, is too unseasonable for all the practical purposes of diagnosis, and in cases in which recovery takes place, is wholly inapplicable. Were it generally impossible to decide between the two types of Continued Fever, during the life of the patient, by distinctive symptoms and laws, the propriety of even a nosological division could not be sustained, and the question of non-identity would be inadmissible. The intestinal lesions now regarded by many as characteristic of the *Typhoid* type, would then be entitled to be considered to be merely complications more or less frequently present in cases of Continued Fever. This is, in fact, the view taken by some writers, who contend that the distinctive symptoms of the two types are not valid, or that they cannot be relied upon for diagnosis. Persons holding this view, as a matter of course, regard the intestinal lesions as incidental events which may, or may not be present; but if they do not practically make the distinction between *Typhus* and *Typhoid*, according to the diagnostic traits deduced from analyses at clinical data, it is plain that they cannot bring the fact of the absence or presence of these lesions in individual cases under their observation to bear on the question of the identity or non-identity of the two types. This, then, is the point which I would enunciate: The determination of the question whether a particular case of Continued Fever be of the *Typhus* or *Typhoid* type, should not rest exclusively on the presence or absence of certain intestinal lesions. It cannot so rest, it is evident, unless the diagnostician, has always the advantage of an autopsy. But, irrespective of this, the basis of the division into the two types is the *ante-mortem* history. Do the two types possess sufficiently distinctive traits to be nosologically separated? If so, the distinction is undoubtedly in no small measure, strengthened by the fact that certain special lesions are peculiar to one of the types. This is a point to be demonstratively established by examinations after death of cases in which the *Typhoid* traits were unequivocally declared during life. So, also, the question whether the lesions peculiar to one of the types are invariably present, is to be settled by repeated examinations after death of cases in which the distinctive traits of that type were exhibited during the progress of the disease. The latter question, as well as the former, should thus be fairly and fully met by an appeal to observation. It has been seen that, in accordance with this view of the subject, it may probably be proved by reference to Louis' own collection of histories, that *Typhoid* fever may exist, and prove fatal without disease of the follicles and mesenteric glands. Other cases have been reported. Prof. Bartlett refers to several, in some of which he appears to think there may have been room for doubt as to the correctness of the diagnosis, and in one striking case in which no suspicion on this score could be entertained, he conjectures that the *Typhoid* lesions had existed, all traces of which disappeared before death; Louis propounds the latter as an hypothesis which would account for the absence of these lesions in the cases supposed by him to be cases of *Typhoid* fever prior to the autopsies, but he prefers to regard them as cases simulating the disease.

The point under consideration must be considered, to say the least, unsettled. Do cases exhibiting during life an assemblage of symptoms sufficient to render it clear that they are cases of the *Typhoid* type of Continued Fever, in the event of their proving fatal, *always* present the lesions which are regarded as characteristic of this type? A farther collection of facts bearing on this inquiry is to be desired. Should the inquiry be settled conclusively (if it be not already) in the negative, then it remains to ascertain, by aggregated observations, in what ratio of *Typhoid* cases these lesions are present.

The connection of this point of inquiry with the subdivisions of Continued Fever is obvious. If it be decided to call those cases only of Continued

Fever *Typhoid*, in which these lesions are present, what shall be done with cases of Continued Fever in which these lesions are absent? shall all be set down as cases of *Typhus*, whether the distinguishing traits of the latter type were present during life or not? The effect of this would obviously be to impair the significance of the symptomatology of the latter type; but as the point of the departure for the question is believed to be wrong, it is hardly worth while to pursue the topic.

There is still another question connected with the intestinal lesions of *Typhoid* fever opening up a subject which it is foreign to my present design to consider at any length, but which calls for a brief passing notice. What relations do these lesions sustain to the disease, i. e. to the Fever? Louis indicates these relations by the expression "*anatomical characteristic.*" By this expression he evidently means to convey the idea of a peculiar, intrinsic element of the disease. He does not regard *Typhoid* fever as simply a follicular enteritis, as has been somewhat pertinaciously reiterated by those not conversant with his writings. A view essentially similar appears to be taken generally by those who look upon these lesions as exclusively belonging to the disease. If the latter opinion be correct, there must exist some special relations between the lesions and the intimate pathological condition constituting *Typhoid* fever; but respecting the character of these relations, it is in vain, with our present pathological knowledge, to inquire. We are, however, not more in the dark here, than with regard to other events in the history of the disease, for example the characteristic eruption. It is difficult to say in what way, and to what extent, the presence of these lesions modifies the course of the disease. They expose the patient to the danger of perforation, and, if extensive and severe, they cannot fail to add to the gravity of the symptoms. Diarrhœa and hæmorrhage are probably due to this cause. It is reasonable to suppose that the more frequent and greater tenderness and meteorism in *Typhoid* fever are owing to the same cause. Convalescence, doubtless, is often retarded by the persisting morbid condition of the follicles and mesenteric glands, and, where the lesions are considerable, they may suffice to destroy life when otherwise the recuperative energies of the system would triumph.

Finally, facts pertaining to distinctive symptoms and lesions, appear to favor the views of those who hold to the non-identity of *Typhus* and *Typhoid* fever. But in arriving at this conclusion a fair allowance should be made for the points of agreement between the two types, not confining the attention wholly to the points of contrast. On instituting a comparison in this point of view, it must be admitted that several important elements are common to both. This is sufficiently apparent on reference to the results of the analytical investigations of cases of the two types. But with reference to this point it is to be remarked that several important elements are common to all fevers, and, indeed, not to fevers alone, but other acute affections. And this remark is so applicable to the group of symptoms common to *Typhus* and *Typhoid*, that it is customary, both in medical writings, and conversation, to apply the term *Typhoid* to denote resemblances occasionally presented in various diseases. Thus we have *Typhoid pneumonia*, remitting or bilious fever with *Typhoid* symptoms, etc. A group of symptoms denoting a morbid condition analogous to that observed both in *Typhus* and *Typhoid* fever, commonly known as the *Typhoid state*, as all practitioners are aware, is recognized in affections which no one thinks of regarding as identical. Community in symptoms which do not exclusively belong to Continued Fever, but are occasionally observed to a greater or less extent, in various affections, although doubtless evidence of relationship between the two types, cannot be considered adequate to establish their identity, while numerous and striking differences point to an opposite conclusion.

To decide that the *Typhoid* and *Typhus* forms of Continued Fever are not identical, is not to determine the kind and degree of relationship existing between them. With respect to the latter topics of inquiry, it is not easy, in the present state of knowledge, to arrive at any very precise ideas. In all fevers, as well as various other affections, there is an inappreciable pathological condition underlying the appreciable phenomena which constitute their natural history. The latter, for the most part, are the objects of investigation, the former lies in the *terra incognita* of science. Were we able to push our researches so far as to grasp the prime essential changes in which disease consists, and thence to trace, synthetically, the train of sequences to which, with our present means of investigation, we are mostly restricted, we might expect to define with exactness the real distinctions and affinities existing among different affections. As it is, we must judge in how far diseases are kindred, or foreign to each other, chiefly by inferential reasoning, based on a comparison of phenomena and their laws; and our conclusions cannot be expected to be more than approximations, more or less remote, to the truth.

That the two types of Continued Fever, *Typhus* and *Typhoid*, are closely related seems altogether probable. They are varied forms of the same branch of the family of Essential Fevers, viz., Continued Fever. They have several important features in common. They are not always, as will be presently seen, readily discriminated. Their application and near resemblances furnish just criteria for estimating relationship, without compromising their separate individuality.

Some questions pertaining to the causation of Continued Fever, as yet open for investigation, have an important bearing on this branch of the subject. It is probably the most rational supposition that this, as well as other essential fevers, always involves, in its production, the operation of a specific morbific agency. Now do *Typhus* and *Typhoid* fever require each a distinct special cause, or may the same specific agency produce either disease according to the direction or modification it receives irrespective of the intrinsic character of the cause? Both types may be propagated by contagion. This seems to be conclusively settled. Now is the contagious miasm furnished by either type capable of giving rise to cases of both types, or cases only of the same type? Another inquiry grows out of that just propounded, viz., does the occurrence of either type of the fever exempt the individual from a recurrence of both types, or only of the particular type experienced? On the latter point some interesting facts are communicated by Dr. Power,* of Baltimore, tending to show that the exemption afforded by having the disease, only extends to the particular type experienced. The facts are, however, too few to authorize the induction of a general law. Observations may hereafter supply positive answers to these and other questions, the connection of which with the present topic, and, indeed, with the subject of the identity or non-identity of the two forms of fever, must be sufficiently apparent.

It is, also, obvious that in proportion as, by continued researches, our knowledge of the natural history of both types becomes more extensive and complete, it may be expected that the distance between them will be more clearly defined.

In concluding these remarks on the identity or non-identity of *Typhus* and *Typhoid* fever, it may be proper to state that the foregoing views differ somewhat from those which the writer has heretofore entertained, and on several occasions expressed.† The study and thought bestowed upon the subject in connection with the analyses upon which the preceding Reports are based, have led to this result. Without presuming that any importance will be attached to the fact, it is mentioned simply because it affords the opportunity of saying that, whether right or wrong, the conclusions are divested of any bias from preconceived opinions.

---

* Bartlett's Treatise on Fever. † Buffalo Medical Journal.

### DIAGNOSIS.

The subject of the diagnosis relates, *first*, to the determination of the *type*' whether *Typhus* or *Typhoid;* and, *second*, to the discrimination of Continued Fever irrespective of type.

*Typhus and Typhoid.* The differential diagnosis, it is obvious, involves those distinctive traits of either type which enter into its natural history. In so far as these are developed by the preceding investigations, they have been briefly stated under the head of *Summary of the distinctive symptoms of Typhus and Typhoid.* A repetition in this connection is unnecessary. In order to decide to which of the two types individual cases are to be assigned, it is requisite, of course, that the practitioner shall be conversant with the historical features by which each is distinguished from the other, as well as competent practically to apply this knowledge by means of correct observation and judgment. Repeated and extended analyses, by enlarging, and defining more accurately our acquaintance with the symptoms and laws of each type, will contribute, more and more, to establish the points upon which the diagnosis is to be based. This remark admits of a general application. Just in proportion to the progress made in knowledge of the natural history of any disease will the means of diagnosis be increased; and our knowledge of the natural history of any disease is enlarged in proportion to the importance of the results developed by analytical investigations. In the majority of cases of Continued Fever falling under observation, it is not difficult to determine whether the form is *Typhus* or *Typhoid.* The eruption suffices to mark the distinction, when this criterion is available, with an occasional exception in which the characters are not well defined, or those of both types are more or less intermingled. And in cases in which the eruption is absent, characteristic features will usually be present sufficiently to warrant a decision as to type. This is true as the general rule, but in some instances, it must be admitted, the question as to type is doubtful, and in a few instances the diagnosis may be quite difficult, and possibly impracticable. It may occur to the reader, in this connection, to inquire, why it is, if the type be generally determinable, that in arranging the cases upon which the preceding Reports are based, so many were excluded from the *Typhus* and *Typhoid* groups, and classed under the head of *Doubtful type.* Of the one hundred cases, *eighteen* were assigned to this division. If this be a just expression of the result of the effort to exemplify, in the writer's experience, the feasibility of diagnosis, it certainly conflicts with the tenor of the foregoing remarks. But it is not a fair inference that in so large a proportion of cases the type is indeterminable. Inasmuch as a prominent object was a comparison of the results of the analytical investigations in the two types, the intention was to exclude any case in which there was the least room for question as to which type it belonged. On reviewing the histories of the cases of *Doubtful type* since commencing to write on this topic, it is pretty clear, in the majority of the cases, to which of the two series they belong, and so it was at the time the classification was made, but the evidence was not deemed complete enough to satisfy the rigorous rule adopted, and in order to avoid the possibility of a confusion which might vitiate the results, they were rejected. A few were excluded in consequence of the histories being imperfect, on account of the patients coming under observation late in the disease. A few were so mild as not to present some of the prominent traits of either type. In a very small number of the cases embraced in the *Doubtful* series, were the characters of *Typhus* and *Typhoid*, inclusive of the eruption, so intermingled as to occasion very considerable difficulty in forming an *opinion* on the subject of diagnosis. In fact, this was true of but *two* cases in both collections. In nearly all the cases of *Doubtful type* the eruption was wanting.

The question has been raised whether the two types of Continued Fever may not be blended in some cases; in other words, whether a kind of *hybrid* affection is not occasionally observed, formed by an union of the characters of *Typhus* and *Typhoid.* Our acquaintance of the relationship which they sustain toward each other is insufficient to predicate thereon any positive opinion with respect to this point. It is a question which, with our present pathological knowledge, must be settled by facts gathered by observation. The histories, in the preceding collections, having a bearing upon this question, are too few, and the facts not sufficiently explicit, to warrant any conclusions.

Another inquiry suggests itself which relates not only to the diagnosis, but to the identity or non-identity of *Typhus* and *Typhoid* fever, viz., Is it not likely that the natural history of Continued Fever is subject to so great mutations incident to the lapse of years, that a transcript of this history based upon observations collected at any particular period will not answer for all time? Is it not probable that the disease has assumed a diversity of phases within a half century or more? That Continued Fever, as well as other affections, at different periods and places may be characterized by striking diversities, is undoubtedly true. The analyses upon which the preceding Reports are based, embracing collections of cases made, for the most part, in two successive years, developed peculiarities incident to each year. In the first collection *parotitis* occurred in several instances, and, in not a single instance in the second collection. The *Typhoid* cases in the second year were distinguished by a remarkably short duration of the febrile career, and by the frequent occurrence of *relapse of fever*, the latter not occurring in a single instance among the cases first analyzed. These are illustrative of variations that may be expected to occur from year to year. Notwithstanding such variations, it may be that, as respects the greater portion of the important elements of the natural history of the disease, there will be found to be a striking uniformity from year to year, and during a long period of time, as well as in different quarters of the world. It is now more than twenty years since the analytical researches upon the *Typhoid* affection, by Louis, were published. The publication of this remarkable work led to the application of the same method of investigation by Dr. James Jackson, of Boston, to the histories of the cases of Continued Fever recorded at the Massachusetts General Hospital during a period of twelve years. The results of the analysis by Dr. Jackson, were found to correspond, in all material points, with those enunciated by Louis, showing the existence of a fever at Paris and Boston having identical phenomena and laws. Not mentioning other investigations, in a small collection of cases occurring at Buffalo, presenting the external distinguishing traits of *Typhoid* fever, we have found, on analysis, a striking correspondence in results with those disclosed at Paris and Boston; and, again, the following year by the analysis of another small collection, the same results are confirmed. The disease, thus, at different periods, and at places widely separated, maintains a strongly marked individuality. Its eccentricities do not compromise a remarkable consistency of character.

As with *Typhoid*, so with *Typhus*, although the latter has not been studied to the same extent as the former. Shortly after the publication of Louis' work, it was ascertained that fever as it prevailed in the British Islands, was not uniformly of the kind described by that distinguished observer. Separating the cases in which the characteristic traits of *Typhoid* were present, from those in which they were absent, the latter were found to exhibit distinctive traits sufficient to warrant their arrangement in a separate class, to which has been restricted the term *Typhus*. Dr. Gerhard, of Philadelphia, was the first to apply, to any considerable extent, the analytical method of determining the natural history of the form of Continued Fever just mentioned. He gives the results of an analysis of a large number of cases occurring at Philadelphia in 1836.*

These results, in so far as the symptomatology of the disease is concerned,

*Am. Jour. of Med. Sciences, vols. 19 and 20.

appear to be, in the main, reproduced wherever, subsequently, the disease has been studied; and we have seen that they are exemplified in the casual cases assembled, on two successive years, in the hospital at Buffalo.

Facts, thus, seem to render it probable that, although the lineaments of the two forms of Continued Fever may be liable to variation to a greater or less extent, the fundamental traits which characterize each, and upon which rest their claims to be considered distinct affections, have as much fixedness as belongs to other diseases. Indeed, judging from the data as yet available, both *Typhus* and *Typhoid* fever are less subject to important changes, from time to time, than other forms of febrile disease, for examples Remittent Fever, Rubeola, and, *especially*, Scarlatina. But with reference to this interesting point of inquiry, multiplied observations and analyses are highly desirable. By this method of appealing to facts, and by this method only, can it be demonstrated, on the one hand, what characters of these, as well as other diseases are preserved from year to year, and age to age; and, on the other hand, the nature and extent of the mutations incident to time or place.

The propriety of resolving all cases of Continued Fever into two forms, *Typhus* and *Typhoid*, and the appropriateness of these terms of designation, are points not yet touched upon. It may be a question whether cases in which the distinctive traits of *Typhus* or *Typhoid* are wanting, or at least not prominent, should not be considered to form a separate class. A certain proportion of the cases of Continued Fever which fell under observation, are of the kind referred to, and this would appear to be true of a consecutive series of cases occurring at particular periods and places, as in the instance of epidemics of what has been described by some authors under the name of *inflammatory* fever. It would obviate a difficulty of diagnosis which must to some extent be felt in practice, to include cases in which it is not easy to recognize distinct evidences of either the *Typhus* or *Typhoid* type under a distinct head.

The expression *simple Continued Fever*, which some writers have employed, might embrace such cases. Or, another course may be pursued, viz., to consider all cases not presenting unequivocal marks of *Typhus*, as belonging to a common series, using, instead of the term *Typhoid*, the phrase *Common Continued Fever*. The latter is the more simple arrangement, and would probably answer every practical purpose.

Objections are frequently made to the terms *Typhus* and *Typhoid*. The former, *Typhus*, has long been applied to denote a form of fever characterized by ataxic symptoms, and especially stupor, to which the etymology of the term refers. The significancy of this term is in a measure impaired by the nosological lines which are now considered to bound the division. While it is still true that ataxic symptoms, including somnolency, or stupor, are apt to be prominent, and earlier developed in *Typhus*, it is also true that these symptoms are not invariably present in a notable degree in that type, while they are not infrequently developed in an equal degree in cases of *Typhoid.* The diagnostic criteria, in short, embrace other traits than those pertaining to the nervous system. Still, as most of the cases to which the term *Typhus* has been heretofore applied, would be so considered in the present acceptation of that term, there is a propriety in retaining it.

The term *Typhoid* is more open to criticism, although it is significant of the fact that frequently in cases of the type which it designates are presented prominent symptoms resembling, and indeed, identical with those observed in *Typhus.* But the term is inappropriate, inasmuch as this significancy only applies to a certain proportion of the cases of the class which it is employed to denote. Besides, the term has become incorporated in medical literature, and common parlance, to denote Typhus-like symptoms occurring in various diseases. If all cases of Continued Fever not exhibiting the diagnostic traits of *Typhus*, are to be considered as embraced in a single nosological group, some other designation than *Typhoid* would seem to be desirable, and this term might still be used to distinguish certain cases which affiliate closely with those of the *Typhus* type. The phrase *enteric fever*, proposed by Prof. Wood, is based in the supposition that all cases are complicated with more or less of the characteristic intestinal lesions. This, as has been seen, cannot be considered established. The expression *Common Continued Fever* is perhaps as unobjectionable as any that, in the present state of our knowledge, could be selected.

*Discrimination of Continued Fever from other affections.* In sections of country in which periodical fevers are more or less prevalent, Remittent and Continued Fevers are to be discriminated from each other. To do this is not always easy, and requires, on the part of the diagnostician, an accurate knowledge of the phenomena distinctive of each disease. It is probable that these affections have been, to a considerable extent, confounded by practitioners. This has arisen, doubtless, in part, from not recognizing the characteristic traits of each, but, in part also from the looseness with which the terms *Typhoid* and *Typhus* have been employed. Cases in which typhus or typhus-like symptoms become developed have been called cases of *Typhus* or *Typhoid* fever without inquiring whether other essential diagnostic criteria were present or not. A *Typhoid condition* is one thing, and *Typhoid fever* another. The first may enter into various diseases. That is to say, the ataxic symptoms which give rise to those external characters of which the term *Typhoid* is significant, such as muttering delirium, coma-vigil, subsultus, prostration, do not exclusively pertain to *Typhoid* fever, and, on the other hand, are not invariably present in cases of *Typhoid fever*. The diagnosis of *Typhoid* fever, in fact, is by no means based solely on the symptoms which it has been, and is still customary to characterize as *Typhoid* symptoms. Hence, the disadvantage of the term *Typhoid* as the title of a particular species of fever. Uncertainty as to the mode in which the term *Typhoid* is applied, has tended, in no small measure, to create distrust of contributions on subjects connected with the disease. Several interesting questions which have occasioned considerable discussion, of late years, remain to be settled by careful and repeated observations made in different portions of the country. One of these relates to the geographical boundaries of *Typhoid* fever — is it affected by climate and topography, and, if so, to what extent, and in what manner? Another is, what are its relations to Remitting fever — do the two fevers prevail together at the same time and place — do they succeed each other — are they convertible the one into the other — may the phenomena of each be intermingled in variable proportions, the two diseases being blended together? The topics involved in these inquiries are exceedingly interesting and important. It does not fall within the scope of my present design to inquire what reliable facts bearing upon them have been contributed; but it must be admitted that they open a field in which there is abundance of room for investigation.

The differential diagnosis of Remitting and Continued Fever, it is clear, is essentially involved in the collection of observations just stated, and others not less interesting and important, for example questions relating to the management of the two fevers. And the differential diagnosis, it is evident, involves those points of contrast which are developed by comparing the two affections as respects the natural history of each. Here, as in all diseases, our available means of diagnosis are proportionate to the minuteness and extent of our knowledge of the natural history of the diseases to be discriminated from each other; and here, as in all diseases, the natural history is to be based on the results of the analytical investigation of recorded cases. This method of investigation has been, as yet, inadequately applied to the study of Remittent Fever. Small collections of cases have been analyzed by Dr. Stewardson, Dr. Gerhard, and Dr. Stille, of Philadelphia, and Dr. Swett, of New York. But to determine the precise relative positions of the phenomena which enter into the symptomatology of the disease more extensive analyses are requisite. It remains to effect for this disease what the labors of

Louis have accomplished for *Typhoid* fever. When this is done, it may be expected that not the least of its advantages will consist in the means of diagnosis being extended and better defined.

Without dwelling on the subject, I will endeavor to give a brief synopsis of the more prominent of the diagnostic traits which, with our present knowledge, appear to be involved in the discrimination of Remittent and Continued Fever. In the great majority of instances in which this discrimination is to be practically made, it will lie between Remitting and *Typhoid* Fever. The following summary, therefore, will embrace the characters distinguishing Remittents from the *Typhoid* type of Continued Fever.

### SUMMARY OF CHARACTERS DISTINGUISHING REMITTENT FROM TYPHOID FEVER.

*Access* oftener abrupt; prodromic symptoms, if present, less prominent, and of short duration. Disease more uniformly ushered in by a *chill;* the latter more pronounced, more apt to be accompanied by *rigor,* and to be longer in duration, in short, more closely resembling the cold stage of an intermittent.

The early stage frequently characterized by *remissions,* consisting in a marked abatement of the febrile movement, recurring with more or less observance of the laws of periodicity observed in intermittents, chills sometimes repeated at the termination of the remissions.

*Quiet, muttering delirium,* and *coma-vigil,* rarely present, and, when present, occurring later in the career of the disease. The delirium, when present early, generally due to high febrile excitement occurring during the height of the exacerbation. Sopor occasionally observed in the exacerbation, probably due to cerebral congestion. *Deafness* very seldom present. *Subsultus, carphologia,* and *great prostration* less apt to occur.

*Nausea* and *vomiting* more constant and prominent symptoms, accompanied by tenderness over the epigastrium. Thirst more urgent. Absence of tenderness in iliac regions, and gurgling and meteorism much less frequently developed in a notable degree. Peritonitis and intestinal perforation do not occur, nor hæmorrhage from the bowels. Diarrhœa not occurring as an element of the disease, irrespective of cathartic remedies or other exciting causes. *Sordes* seldom observed.

Cough, with sibilant and sonorous rales, less constant. Pneumonitis less apt to occur as a complication. Epistaxis not an element of the disease.(?)

No eruption of rose spots. Sudamina less frequent in occurrence. Yellowness of conjunctiva and surface of body oftener observed. Eschars more infrequent. Congestive redness not so uniform or marked.(?)

Physiognomy less characteristic. A stupid, besotted expression, or an absence of all expression more rarely observed.

No predilection for autumnal months; more apt to occur during the heat of summer.

Does not evince preference for young subjects.

Not infectious nor contagious.

Prevails only in miasmatic regions, or attacks those who have been exposed to miasmatic influences.

Frequently ends in intermittent fever, and persons having experienced the disease are subject to subsequent attacks of intermittents.

The distinctive traits comprised in the foregoing summary are sufficient to denote a form of fever quite different from either type of Continued Fever. They suffice also for practical diagnosis in the great majority of cases, even although the most striking characteristic of Remittent Fever which suggested the name by which it is distinguished, to wit, the *remissions*, may be absent, or have ceased to recur prior to the patient coming under observation. That *Remittent* and *Typhoid* Fevers have been confounded, more or less, by practitioners, may be owing to various causes. In districts in which periodical fevers are the predominant form of febrile disease, the study of the characteristic phenomena of *Typhoid* fever is apt to be neglected, and this form of fever is accordingly overlooked. Moreover, it remains to be determined whether, and to what extent, the two fevers are capable of being blended, and mutually convertible.

The traits involved in the differential diagnosis, however, will doubtless be more accurately determined, and possibly new points of contrast developed, when the natural history of each disease, but especially of Remittent fever, shall have been perfected by further analytical researches.

Continued Fever is to be distinguished from inflammations, and local affections accompanied by febrile movement. There is greater liability of mistaking Continued Fever for diseases seated in particular organs, than the reverse; but an error of diagnosis, in either way may be committed. So far as my knowledge and experience extend, Continued Fever, not recognized as such, may be considered to be *Encephalitis*, *Pneumonitis*, and *Enteritis*. I have heard of instances in which *Tuberculosis*, with tuberculous fever, were set down as cases of Continued Fever. The three affections just named viz., encephalitis, pneumonitis, and enteritis, may actually be present in connection with Continued Fever, the error then consisting in regarding them as *primary*, when they are *secondary* diseases; or the diagnosis may be predicated on prominent symptoms referable to the head, the chest, or the abdomen, when inflammation affecting either of these anatomical divisions, does not exist even as a complication of the fever.

The discrimination involves several general principles which are applicable alike to each of the affections. *First*, if the disease be Continued Fever, in the majority of cases it will have been preceded by an access of several days' duration. This is a feature somewhat characteristic. *Second*, the evidences of fever will probably have been declared prior to the development of the local affections, or of the prominent symptoms which lead to the supposition of the existence of the local affections. *Third*, the local symptoms referable to the organs supposed to be the seat of disease, and which may actually be affected secondarily, although they may be prominent, are yet disproportionately so when compared with the general symptoms—prostration, disturbance of intellect, etc. *Fourth*, symptoms characteristic of fever, and which do not belong to the history of the local affections, may be expected to be present, such as the eruptions, epistaxis, congestive redness, meteorism, abdominal tenderness, etc. *Fifth*, symptoms are absent which it would be expected should be observed if the local affections were present, or present as primitive affections.

Some of the principles in the foregoing list may be available in cases which come under observation after the fever has advanced more or less in its career, and when it is difficult or impossible to obtain an authentic account of the previous history. The practitioner is more liable to be deceived in such cases, than when the disease has been under observation from the beginning. But even with this disadvantage, if conversant with the distinguishing characters of Continued Fever, and practically familiar with the local affections named, the discrimination should not often prove difficult. Without devoting to this branch of the subject extended consideration, I will offer a few remarks on the application of the above principles to each affection separately.

*Encephalitis.*—Active, persistent delirium, preceded by cephalalgia in some instances, and, in other instances, a state of profound somnolency, or coma, may lead the practitioner to think that encephalic inflammation is the original disease. The collections of cases upon which were based the preceding reports, contained illustrations of prominent cerebral symptoms in both these forms. Of the cases thus characterized, in which post mortem examinations were made, no evidences of encephalic inflammation were found. The cerebral symptoms here referred to, therefore, however prominent, do not necessarily denote that encephalitis exists even as a complication of the fever. Putting aside the diagnostic evidence to be derived from the development and early stage of the disease, and also the consideration that acute encephalitis, in the adult, except as the result of mechanical injuries, is an exceedingly rare affection in this climate, the histories of each of these cases show, on the one hand, the presence of characters distinctive of *Typhoid* Fever, and, on the other hand, the absence of symptoms distinctive of *Encepalitis.* In the first category are meteorism, tenderness in the iliac regions, diarrhœa, etc. In the second are increased sensibility to light and sounds, corrugation of eyebrows, vomiting, constipation, etc. The former were present when they should not have been, had the disease not been Continued Fever; and the latter were absent when it should have been otherwise had acute encephalitis existed. Here, again, it is seen how diagnosis is based on points of contrast evolved by the study of the natural history of diseases. Some of the more obvious and striking symptoms may be common, in certain cases, to different affections, while the less conspicuous and minor details suffice to mark the distinction, and thus obviate errors which would be inevitable without due attention to the latter. Practical skill in diagnosis, thus, is commensurate, not alone with the tact and sagacity of the practitioner, but with the extent of his acquaintance with the historical features of diseases.

*Pneumonitis.* It is to be presumed that, in cases in which it is a matter of doubt whether the disease be Continued Fever or pneumonitis, the latter exists as a complication. To decide whether a pneumonitis be a *secondary* or *primary* affection, may sometimes be attended with real difficulty, arising from the fact that when primary it is sometimes associated with some of the more prominent of the symptoms of *Typhoid* or *Typhus* fever, forming what is called *Typhoid Pneumonia.* We may have the delirium, prostration, etc., which constitute the *Typhoid state,* in connection with pneumonitis, occurring irrespective of *Typhus or Typhoid* fever. It is to be remarked, however, that cases of what is called *Typhoid Pneumonia* are, probably, not infrequently cases of *Typhoid* or *Typhus* fever, with pneumonitis as a complication. There is reason to suppose that the two affections have been confounded, to a greater or less extent, owing to the diagnostic lines not having been, until lately, distinctly drawn. If this remark be true, *Typhoid Pneumonia* does not occur so often as would be inferred from past writings.

In the differential diagnosis the early history is especially important, for it probably is rarely the case that pneumonitis, occurring as a complication of Continued Fever, becomes developed from the commencement of the febrile career. If the disease has been observed from the first, therefore, it will be evident that the local affection is consecutive to the fever. Frequently it does not occur until late in the progress of the disease. When this is the case, the inflammation is of a low grade, and would be likely to escape, not only detection, but suspicion, if physical exploration were not employed. The physical signs, moreover, are less developed than when the affection is primary, or, at least, this remark is applicable to the special auscultatory sign — the crepitant rale.

To determine the relation between the gravity of the general symptoms and the local affection, is an important point in the diagnosis, inasmuch as by means of physical exploration the extent of the pneumonitis may be accurately gauged. A striking disproportion between the general condition and the local inflammation should lead to the presumption that the latter is a complication in cases which come under observation after the febrile career has advanced more or less, and we have not the advantage of the previous history. But, in addition to the evidence derived from these two sources, we are to look for special symptoms characteristic of Continued Fever, and which do not enter into the natural history of pneumonitis, such as an eruption, the abdominal symptoms, epistaxis, etc. A sufficient number of the latter, it may be expected, will be present to determine the diagnosis, even when it is required to be made late in the disease, without our being able to ascertain whether, or not, the fever had precedence of the pneumonitis, and without reference to a disparity between the local and the general symptoms.

*Enteritis.* The prominence of diarrhœa and other abdominal symptoms leads to the error of supposing the disease is enteritis. Cases in which this error is committed are of the *Typhoid* type, and follicular enteritis, the characteristic complication of this type, is, of course, present. The diarrhœa and other abdominal symptoms may have been prominent from the commencement of the disease, this being true of some cases of *Typhoid* fever. The diagnostic principle of the fever taking precedence of the local affection, would then fail in its application.

The disproportion between the local and general symptoms is a point here specially applicable.

The presence of the *Typhoid condition* would not be looked for in connection with primary enteritis.

The characteristic traits of *Typhoid* fever, viz., eruption, meteorism, epistaxis, etc., traits not belonging to the natural history of ordinary enteritis, possess not less significance than when the differential diagnosis involved the two affections previously considered.

The error of mistaking Continued Fever for local affections is oftener committed when children are the subjects of the disease. The diagnosis of infantile Continued Fever is less easy than when patients are adults. It is sometimes difficult, in children, to appreciate some of the distinctive symptoms, such as the state of the mind, the degree of abdominal tenderness, etc. The rule of the proportion between local and general symptoms is not the same in children as in adults. The constitutional disturbance is greater from a less amount of local disease in the former. Moreover, some of the distinctive traits of Continued Fever appear to be oftener wanting. This is probably true with regard to the eruption. There is reason to suppose that cases of Continued Fever occurring in childhood are not unfrequently regarded as cases of encephalitis and enteritis. The more frequent occurrence of both these affections in early life conduces to these errors of diagnosis. This is a branch of the subject possessing not a little practical importance, since the instance of an incorrect discrimination on the treatment can hardly fail to be pernicious. I shall content myself, however, with this brief allusion to it. An analytical investigation of a collection of cases of infantile Continued Fever is much to be desired, with a view, among other useful ends, to determine in what manner, and to what extent, the disease is modified by circumstances incident to childhood.

